# Respiratory Sinus Arrhythmia in Children—Predictable or Random?

**DOI:** 10.3389/fcvm.2021.643846

**Published:** 2021-05-20

**Authors:** Paulina Lubocka, Robert Sabiniewicz

**Affiliations:** Department of Pediatric Cardiology and Congenital Heart Disease, Medical University of Gdańsk, Gdańsk, Poland

**Keywords:** growth, pediatrics, blood pressure, cardiovascular, electrocardiography, heart rate

## Abstract

**Background:** Respiratory sinus arrhythmia (RSA) is associated with better health in children.

**Aim:** The study was conducted to analyze the trajectory of RSA in 10-year-olds.

**Methods:** A follow-up study on 120 healthy children (62 boys) aged 10.7 ± 0.5 years consisted of a standard 12-lead electrocardiogram, measurements of height, weight and blood pressure. The protocol was repeated after 3 years. Assessment of RSA based on semi-automatic measurements of RR intervals included: the difference between the longest and shortest RR interval duration (pvRSA), the root mean square of differences between successive RR intervals (RMSSD), the standard deviation of the RR interval length (SDNN) and their equivalents corrected for heart rate (RMSSDc and SDNNc).

**Results:** A the first visit 61.7% of children presented with RSA; 51.7% 3 years later. 23.3% of them had RSA only on the first examination; 13.3% only on the second one. The pvRSA, RMSSD, and SDNN measured in 2019 did not differ significantly from their 2016 equivalents (*p* > 0.05). The decline in RSA defined by RMSSD was noted in 52.5% of children and in 54.2% when defined by SDNN. The corrected values decreased in 68.3 and 64.2% of the participants for RMSSDc and SDNNc, respectively. The students with RSA at both visits had lower heart rate (*p* < 0.001) and systolic blood pressure (*p* = 0.010) compared to those with rhythmic electrocardiograms.

**Conclusions:** RSA in children is changeable, though its measurable indices should be adjusted to heart rate.

## Introduction

Respiratory sinus arrhythmia is a physiological phenomenon that results from the parasympathetic nervous system's regulation of the heart's conductive system (1). It is known to be more pronounced in children and young adults than in older subjects, however, the data on the exact time of physiological disappearance and clinical implications of this phenomenon are inconsistent (2–4). Gradual decrease in the resting heart rate observed in children is a source of bias in the assessment of the RSA trajectory across time as the elongation of RR intervals permits their higher variability (5–7).

From a technical standpoint, the RSA is an important component of high frequency heart rate variability (HRV), which is considered a marker of physical and psychosocial well-being of an individual. Low values of HRV indices are observed in patients with a variety of lifestyle diseases: obesity (8–10), hypertension (11, 12), insulin resistance (13), coronary artery disease (14), etc. On the other hand, taking up physical training and a reduction of body mass in the overweight results in an increase of their HRV (15). What is more, high HRV is a positive prognostic factor in adult patients after heart transplantation (16) and myocardial infarction (17).

Considering the above-mentioned implications of the HRV level, it can be hypothesized that the absence of RSA in children or its early disappearance is a risk factor for cardiovascular disease in the future. Early identification of these patients with a diagnostic tool as simple as a resting electrocardiogram, implementation of prophylaxis and regular screening would be beneficial for their future cardiovascular health.

The study aimed at providing qualitative and quantitative assessment of RSA in preadolescent children over the course of a 3-year observation period in relation to their lifestyle and somatic growth.

## Materials and Methods

The study was conducted as part of the yearly SOPKARD-Junior (18) program aimed at primary prevention of lifestyle diseases. It was originally addressed to 5th grade students (10–11 years of age) in September 2016 and repeated after 3 years in September 2019. The program included all children from the town of Sopot (about 36,000 inhabitants) with a written informed consent granted by their parents. In September 2016, 164 students participated in the program, 131 (79.9%) of which were present again during the second edition. One hundred twenty students with correct ECG recordings obtained in both editions were included in the study. We excluded 11 participants, 6 of them for heart rhythm disturbances on one of the recordings and 5 for school absence on the day of the examination. None of the subjects had signs of a substantial heart defect or cardiomyopathy on transthoracic echocardiography. All the procedures included in the programme, apart from echocardiography, took place at schools on regular school days between 8 a.m. and 1 p.m. We ensured that the participants refrained from physical activity nor did they take tests within 2 h prior to medical evaluation. On the examination day, the students' anthropometric parameters (height, weight, waist, and hip circumference) and their blood pressure were measured. Subsequently, the participants were transferred one by one to a separate quiet room, where a 12-lead electrocardiogram was performed. Each of the children received a simplified oral information on the scope and technique of examination. They were asked to lay down in supine position while the electrodes were placed on their chest and limbs. The respiratory rate was not imposed. In case the student presented a voluntary breathing pattern (deep, regular breathing), the investigator disturbed their attention with a neutral conversation. After 60 s measured from the onset of spontaneous breathing, a 10-s piece of ECG was recorded. Mortara Eli 280 instrument was used, with the chart speed of 25 mm/s and a calibration of 10 mm/mV. All tests for each year were performed by one physician, who assessed their technical quality straightaway. In case it was not satisfactory, the ECG was repeated. All files were converted to digital format. For each recording, all the RR intervals were measured after magnifying the image by 200%. We used a digital measuring tool with the accuracy of 0.01 mm from Adobe Acrobat Reader DC software. All measurements were taken by one person. The study design was approved by the local bioethics committee.

Z-scores for height, weight and BMI were calculated according to growth reference values from the OLAF study (19). The following indices were calculated based on the RR measurements:

pvRSA – the difference between the longest and shortest RR interval throughout the recording in ms
RMSSD – the root mean square of differences between successive RR intervals
SDNN – the standard deviation of the RR interval length.

RMSSD and SDNN were corrected for heart rate (RMSSDc and SDNNc) according to the exponential model used by van den Berg et al. (20).

The electrocardiograms were classified into two groups according to their pvRSA level. Recordings with the pvRSA above 160 ms were included in the “RSA” group and the remaining ones were considered rhythmic. In each screening the students were assigned to the RSA or rhythmic group. We then conducted a comparative analysis of anthropometry, laboratory test results and the level of physical activity between participants with RSA at both screenings and their peers who had two rhythmic electrocardiograms recorded.

The statistical analysis was performed in TIBCO Software Inc.'s version 13 of Statistica (2017). We used the Shapiro-Wilk test to determine normal distribution of data. Because of the lack of normal distribution in multiple parameters, the Mann-Whitney *U*-test was used for comparative analysis of groups and the Wilcoxon test was used for related samples. Spearman coefficients were calculated as determinants of correlations between variables.

## Results

One hundred twenty students (62 boys and 58 girls) were included in the study. The age of the participants at the time of the first evaluation was 10.7 ± 0.49 years with no significant difference between sexes. The demographic and anthropometric data of the study population were assembled in Table 1.

**Table 1:** Descriptive statistics of the study population presented as mean (SD) for boys and girls separately.

	**Total**		**Boys**		**Girls**	
	**M**	**SD**	**M**	**SD**	**M**	**SD**
HR (bpm)	81.3	12.47	79.2	10.98	83.6	13.64
Δ HR (bpm)^*^	−7.0	13.04	−3.1	11.67	−11.2	13.20
Height (cm)	148.1	7.97	148.5	8.02	147.7	7.96
Δ height (cm)^**^	18.1	4.75	20.3	4.12	15.9	4.35
Weight (kg)	39.9	9.83	40.4	10.11	39.4	9.59
Δ weight (kg)	16.8	5.93	17.7	6.81	15.9	4.76
BMI (kg/m^2^)	18.0	3.12	18.1	3.22	17.9	3.05
Δ BMI (kg/m^2^)	2.4	1.90	2.2	2.08	2.7	1.68
Waist cx (cm)^*^	64.7	8.75	66.7	9.45	62.8	7.59
Δ Waist cx (cm)	6.2	6.77	5.9	7.04	6.4	6.55
Hip cx (cm)	77.3	8.31	77.5	8.66	77.0	8.02
Δ Hip cx (cm)	12.5	8.21	12.3	8.64	12.6	7.84
WHR^**^	0.8	0.06	0.9	0.06	0.8	0.05
Δ WHR	−0.0	0.10	−0.0	0.08	−0.0	0.12
SBP (mmHg)	108.7	8.99	107.9	8.65	109.6	9.31
Δ SBP (mmHg)	6.9	10.07	9.0	9.82	4.9	9.99
DBP (mmHg)	64.9	7.43	63.6	7.12	66.2	7.56
Δ DBP (mmHg)	1.8	7.73	3.1	7.90	0.5	7.41
MBP (mmHg)	79.5	7.05	78.4	6.65	80.7	7.31
Δ MBP (mmHg)	3.5	7.15	5.1	7.01	2.0	7.03
TC (mg/dl)	181.0	27.20	183.3	27.73	178.6	26.67
Δ TC (mg/dl)^*^	−22.7	18.25	−26.6	19.93	−18.6	15.45
TAG (mg/dl)	74.7	33.90	71.2	34.12	78.5	33.59
Δ TAG (mg/dl)	2.2	36.97	3.9	33.09	0.3	40.94
Glc (mg/dl)	89.2	8.40	90.0	8.63	88.4	8.16
Δ Glc (mg/dl)	−0.4	10.86	−0.3	13.64	−0.5	6.95
HDL (mg/dl)	64.1	12.50	64.1	12.96	64.1	12.12
Δ HDL (mg/dl)^**^	−7.9	8.05	−11.4	7.22	−4.2	7.24
Hgb (g/dl)	13.6	0.68	13.6	0.73	13.5	0.62
Δ Hgb (g/dl)^**^	0.8	2.21	1.3	2.08	0.2	2.23

*Upper rows stand for initial values of distinct parameters, whereas lower rows present their change throughout the observation period*.

*HR, heart rate; Δ, change of the parameter throughout the observation period; cx, circumference; WHR, waist/hip ratio; SBP, systolic blood pressure; DBP, diastolic blood pressure; MBP, mean blood pressure; TC, total cholesterol; TAG, triglycerides; Glc, fasting glucose; HDL, high density lipoproteins; Hgb, haemoglobin*.

* *Significant difference between boys and girls (p < 0.05)*.

** *p < 0.001*.

**Figure 1:**
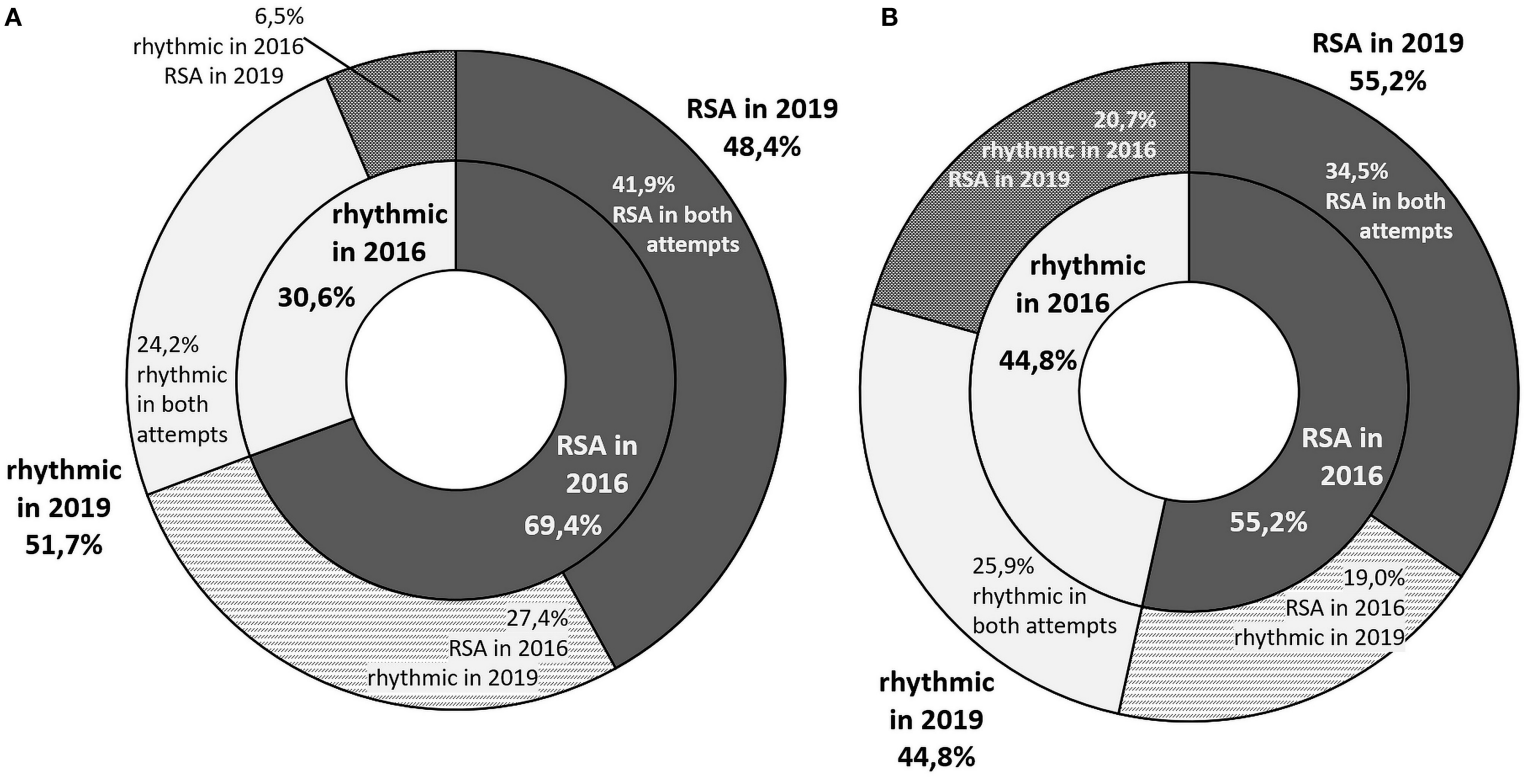
A graphic representation of the proportion of students with respiratory sinus arrhythmia in both visits. The inner ring represents the health status in year 2016, and the outer ring - in 2019. The participants whose status changed across time are represented with intermediate colors in the outer ring **(A)** graph stands for boys and **(B)** one for girls.

At the time of first visit 61.7% of the participants (69.4% of boys and 55.2% of girls) were included in the RSA group, while 3 years later it was 51.7% (48.4% of boys and 55.2% of girls). 23.3% of children (27.4% of boys and 19% of girls) initially placed in the RSA group had a rhythmic heart rate on the second examination. An opposite situation (RSA only on the 2nd visit) occurred in 13.3% of the participants (6.5% of boys and 20.7% of girls) (Figure 1).

Quantitative analysis of the RSA indices revealed that the pvRSA, RMSSD and SDNN values measured in 2019 did not differ significantly from their 2016 equivalents. Parameters with heart rate correction (RMSSDc and SDNNc) differed substantially in female students. There were no significant differences regarding RSA indices between boys and girls in either of the two visits (Table 2). We observed significant correlations between all of the analyzed parameters. High degree of correlation was noted for uncorrected values and SDNNc, moderate degree for RMSSDc and low degree for the same measurements taken during both visits (Supplementary Table 3).

**Table 2:** RSA indices at both visits.

	**2016**		**2019**		***p*^**a**^**	**2016 boys**		**2019 boys**		***P*^**a**^**	**2016 girls**		**2019 girls**		***P*^**a**^**
	**M**	**SD**	**M**	**SD**		**M**	**SD**	**M**	**SD**		**M**	**SD**	**M**	**SD**	
pvRSA	196.5	93.0	191.9	107.7		202.7	86.8	199.1	123.0		189.9	99.62	184.2	88.94	
RMSSD	61.1	35.5	64.3	43.3		64.4	35.4	67.5	50.3		57.6	35.62	60.8	34.48	
SDNN	63.3	32.6	63.2	38.9		65.1	29.1	66.0	46.1		61.3	36.12	60.2	29.48	
RMSSDc	115.1	56.3	95.6	53.0	^**^	112.1	46.5	103.4	60.2		118.2	65.39	87.3	43.01	^**^
SDNNc	43.4	30.0	51.0	39.6	^*^	46.5	29.9	52.7	46.0		40.2	30.15	49.1	31.75	^*^

a *Results of the Wilcoxon's test for all students in year*.

* *Significant difference between samples (p < 0.05)*.

** *p < 0.001*.

**Figure 2:**
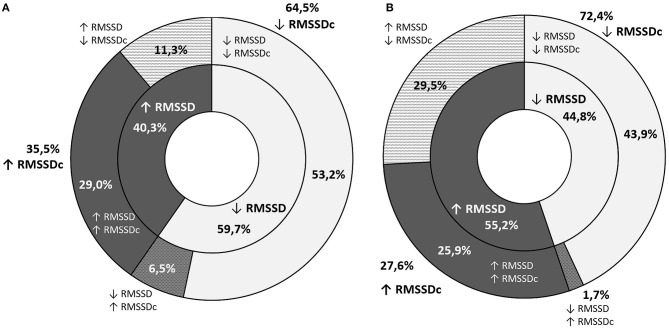
A graphic representation of the proportion of students with an increase or decrease in RMSSD (inner ring) and RMSSDc (outer ring) throughout the observation period **(A)** graph stands for boys and **(B)** one for girls.

**Figure 3:**
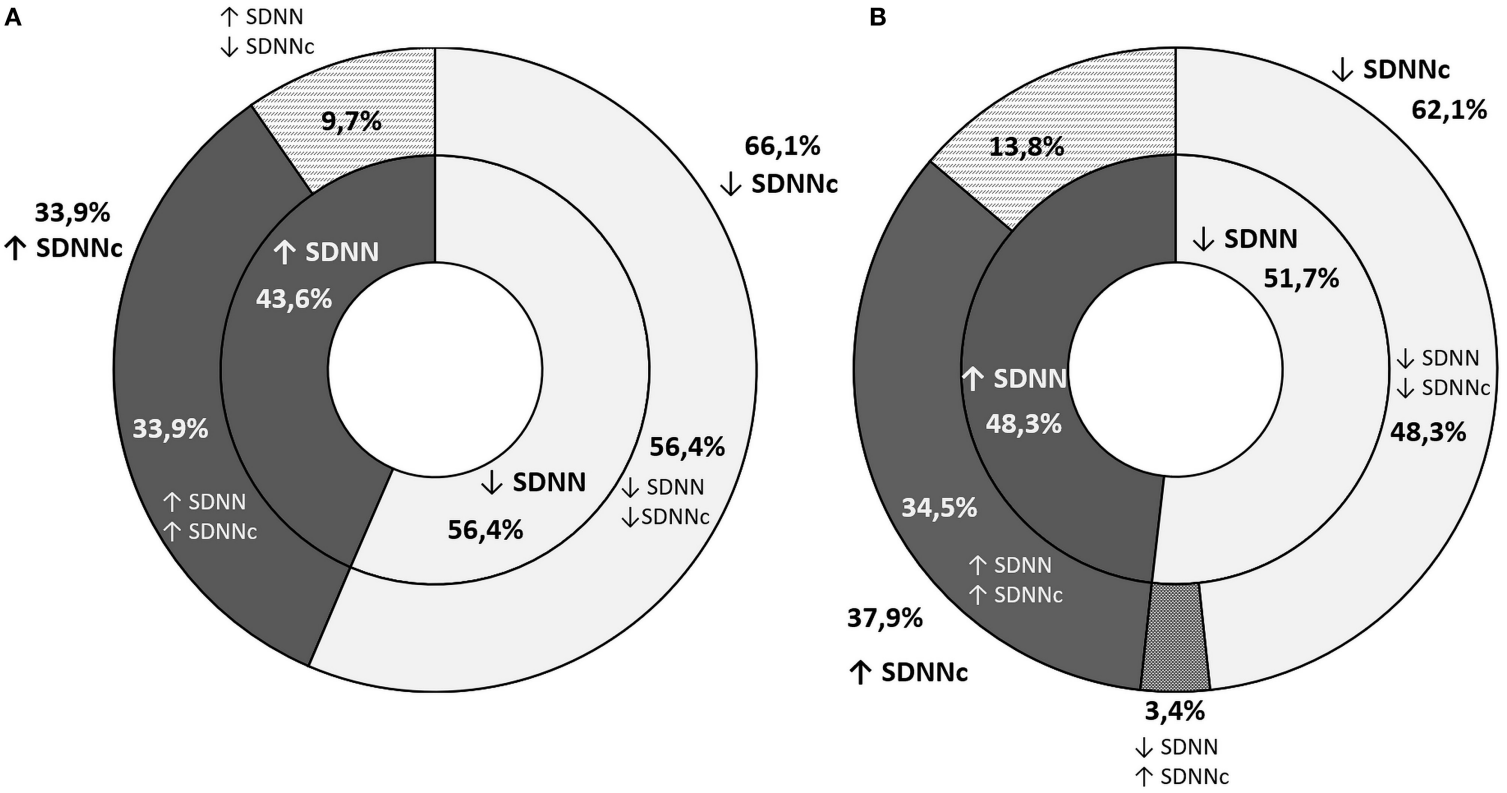
A graphic representation of the proportion of students with an increase or decrease in SDNN (inner ring) and SDNNc (outer ring) throughout the observation period **(A)** graph stands for boys and **(B)** one for girls.

The decline in RMSSD throughout the observation period was noted in 52.5% of the participants (59.7% of boys and 44.8% of girls). Despite an increase in RMSSD, its corrected equivalent was lower after 3 years in 11.3% of boys and 29.5% of girls (Figure 2). In these cases, greater RMSSD was a result of a significantly lower heart rate in the second screening. The opposite situation was noted in a smaller number of cases (4.2%: 6.0% of boys and 1.7% of girls). Changes in the SDNN across time were similar with a smaller discrepancy between boys and girls (Figure 3).

**Figure 4:**
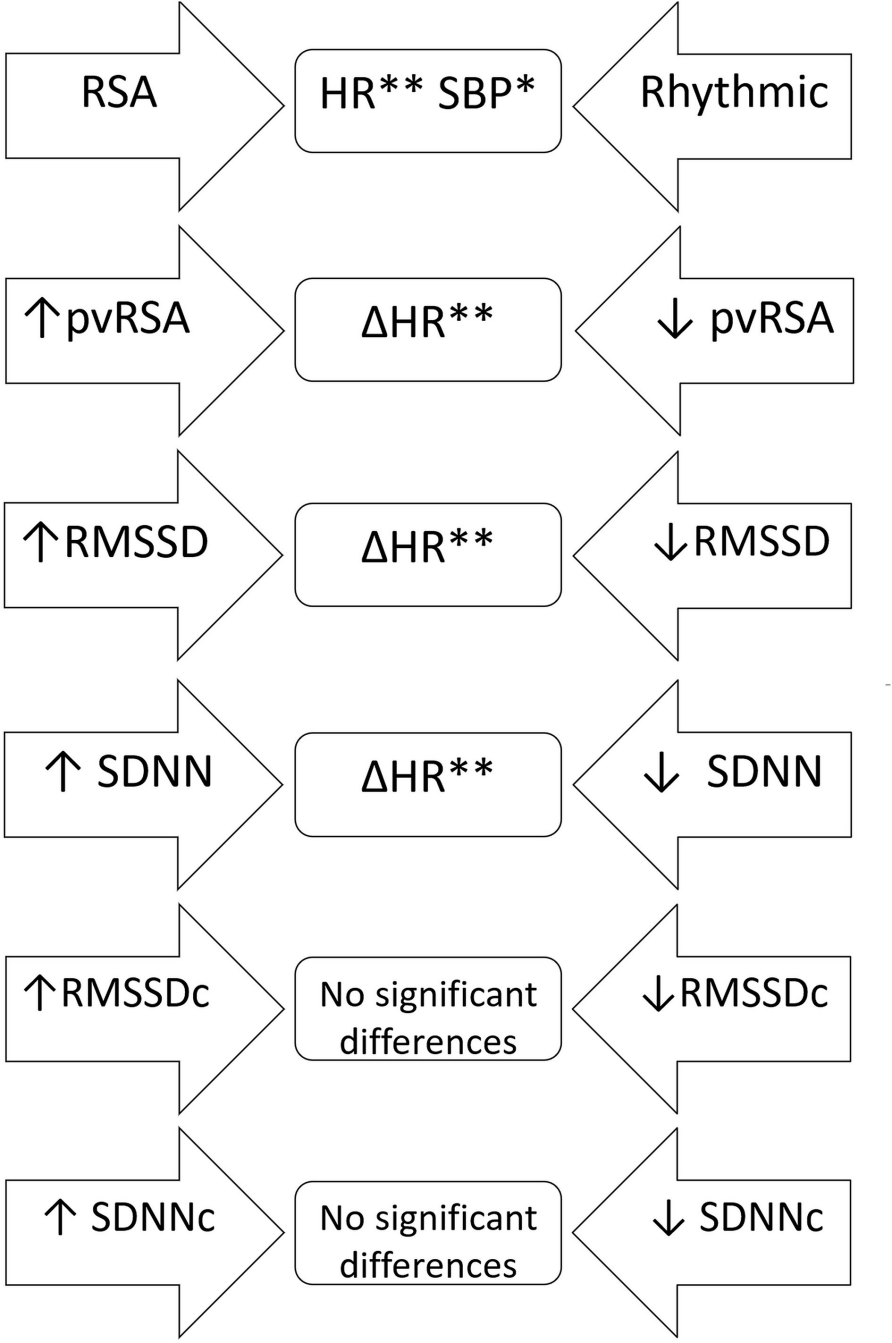
A scheme of comparative analysis of independent samples regarding anthropometric and laboratory variables as well as their changes across time in relation to the trajectory of various RSA indices. The arrow-shaped fields represent compared subgroups; variables, for whom the differences were statistically significant were listed in the middle part. RSA, students with respiratory sinus arrhythmia at both visits; Rhythmic, students with rhythmic heart rate on both electrocardiograms; ↑, increase in a distinct variable across time; ↓, decrease in a distinct variable across time; Δ, change of the parameter throughout the observation period. *p < 0.05; **p < 0.001.

The students with RSA diagnosed at both visits had significantly lower heart rate and systolic blood pressure compared to those with two rhythmic electrocardiograms. An analysis of subjects with an increase or decrease in RSA indices proved no significant differences in terms of their initial anthropometry, blood pressure and lab test results or their change across time. Students who experienced a decline in the uncorrected RSA markers (pvRSA, RMSSD, SDNN) were characterized by a smaller HR drop compared to the remaining subjects, however no statistically significant differences in the initial HR were observed. The correction of RMSSD and SDNN for heart rate removed the above-mentioned pattern, proving no additional discrepancy between the analyzed subgroups (Figure 4).

## Discussion

Our study highlights some important matters in the field of respiratory sinus arrhythmia in children. The first issue is the absence of a precise, quantitative definition of RSA in literature. The discussed phenomenon is mainly described as a continuous variable with no cut-off points. Even though such an approach appears reasonable in the physiological context (RSA as a marker of parasympathetic activity), it impedes communication between specialists who are not familiar with the topic of HRV. In our study, we applied the general criterion of arrhythmia (pvRSA > 160 ms), which divided the population into two subgroups efficiently, avoiding complicated calculations. In our opinion, this was an appropriate method for the present study, however in younger children with a higher heart rate, it would not be applicable.

Another important issue is the well-known relation between HR and its variability (6, 21). The increase of HRV accompanying the HR drop is due to mathematical and physiological factors (both phenomena remain under the control of the parasympathetic nervous system) (22). However, some factors have been reported to affect HR and HRV independently. One of them is age (20). In our study, we observed a decline in HR in the 3-year follow-up period, which is consistent with commonly used reference values (23, 24). Despite the significant HR drop, RSA indices did not change substantially – and decreased effectively when corrected for HR. According to recent publications, standardization of HRV based on momentary HR is particularly important in children, since their heart rate is not stable across time as compared with adults (5). The gradual decline in RMSSDc and SDNNc in the pediatric population was reported by other authors (5, 20). On the other hand, Silvetti et al. (25), who analyzed children's HRV in the form of rough variables, reported their increase in the first 10 years of life followed by a steady decline till adulthood. In our study, we presented both corrected and uncorrected values to provide a complete picture of the RSA trajectory in the population. We assume that standardization of HRV in children is useful, especially when comparing subjects from different age groups.

**Figure 5:**
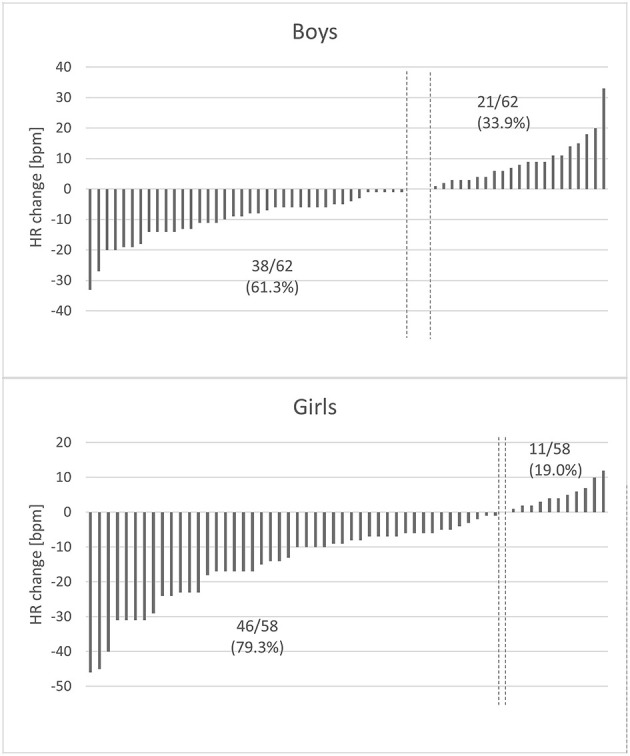
Changes in heart rate in relation to sex. The y-axis represents heart rate difference (the 2019 HR value – the 2016 HR value), with each column representing one child. The x-axis represents study participants arranged by HR difference. The vertical dashed lines symbolize class limits between participants whose HR increased and decreased over the course of our study. HR decreased in 79.3% of girls and only in 61.3% of boys. There was a substantial difference between the degree of HR change observed in boys and in girls (*p* = 0.002).

There are multiple studies on the influence of other factors on RSA in various age groups. Not only do HR and age affect it, genetic predisposition greatly influences the degree of RSA as well (26, 27). Other variables are less impactful, but reports on a possible link between lifestyle diseases and RSA impairment appear to be promising. So far, results of these studies are inconclusive: Tascilar reported reduced HVR in children with obesity as compared to their age-matched controls (13), Quilliot noticed no significant difference in terms of BMI, but confirmed the negative influence of insulin resistance and age on HRV (28), Rabbia attributed the discrepancy in the degree of RSA among pediatric patients with excessive body mass to the duration of obesity (29). Pal noticed a significant inverse correlation between RSA in young pre-hypertensives and their cardiovascular risk (12), whereas in the African-PREDICT study (30) HRV indices were not affected by elevated systolic blood pressure. These contrary findings might be the result of differences in group assortment or applied methodology (length of the recording, analyzed variables and correction for HR). Holter monitors and wearable devices, which provide detailed information on heart's activity throughout a long period of time, can be useful in the thorough analysis of the RSA pattern. However, long ECG recordings are affected by multiple factors, such as level of physical activity, sleep quality, nutrition and psychological stressors on the day of examination, which renders their standardization more difficult (31). Considering that, studies on RSA in children should be revised with caution. The current study did not confirm the hypothesized association between indices of adiposity and a sedentary lifestyle with RSA based on short ECG recordings in early adolescence. Neither an increase or decrease in RSA markers was indicative of changes in anthropometric and laboratory variables. An interesting phenomenon observed in our study is the sex-related difference in the change of RMSSD. According to current knowledge, the impact of sex on RSA is minimal with its values slightly higher in women since early adulthood (20). Our study is in line with the findings of Gasior (5), who reported differences between boys and girls regarding RMSSD and SDNN, but not their corrected equivalents. Despite the fact that none of the variables (corrected or uncorrected) differed between sexes, more girls experienced a decline in HR than boys (Figure 5) and their HR at the 2nd visit was significantly lower (*p* < 0.001) than the initial one. The above-mentioned difference might be attributed to a different maturation pattern in boys and girls and can be subject to further evaluation.

In conclusion, respiratory sinus arrhythmia in early adolescents is a changeable phenomenon. Its presence is highly dependent on momentary heart rate and probably situational. The age-related decrease of high frequency HRV in children aged 10–13 is partly concealed by their decreasing heart rate. Standardization of HRV indices in this group is though recommended to prevent contradictory results of future studies. RSA measurement on a 10-s electrocardiogram and its change throughout a 3-year observation period does not assess predisposition to non-communicable diseases in pre-adolescents adequately. Possibly, increasing frequency of the examinations could provide more coherent information and determine the high-risk group.

## Data Availability Statement

The raw data supporting the conclusions of this article will be made available by the authors, without undue reservation.

## Ethics Statement

The studies involving human participants were reviewed and approved by Independent Bioethics Committee for Scientific Research at Medical University of Gdańsk. Written informed consent to participate in this study was provided by the participants' legal guardian/next of kin.

## Author Contributions

Material preparation, data collection, and analysis were performed by PL. The first draft of the manuscript was written by PL and revised by RS. All authors contributed to the study conception and design, read and approved the final manuscript.

## Conflict of Interest

The authors declare that the research was conducted in the absence of any commercial or financial relationships that could be construed as a potential conflict of interest.
