# Supplementary material for: Respiratory Sinus Arrhythmia in Children—Predictable or Random?
Source: Front Cardiovasc Med. 2021 May 20;8:643846. doi: 10.3389/fcvm.2021.643846 (PMC8172810; doi:10.3389/fcvm.2021.643846)
Supplement: Supplementary file 1 [file Table_1.DOCX]

Supplementary Material

# Supplementary Tables

|  | **pvRSA** | **RMSSD** | **SDNN** | **RMSSDc** | **SDNNc** |
| --- | --- | --- | --- | --- | --- |
|  | **2016** | | | | |
| **pvRSA** |  | 0.887 | 0.971 | 0.617 | 0.804 |
| **RMSSD** | 0.887 |  | 0.904 | 0.678 | 0.660 |
| **SDNN** | 0.971 | 0.904 |  | 0.614 | 0.806 |
| **RMSSDc** | 0.617 | 0.678 | 0.614 |  | 0.832 |
| **SDNNc** | 0.804 | 0.660 | 0.806 | 0.832 |  |
|  | **2019** | | | | |
| **pvRSA** |  | 0.922 | 0.983 | 0.745 | 0.901 |
| **RMSSD** | 0.922 |  | 0.923 | 0.791 | 0.797 |
| **SDNN** | 0.983 | 0.923 |  | 0.699 | 0.884 |
| **RMSSDc** | 0.745 | 0.791 | 0.699 |  | 0.864 |
| **SDNNc** | 0.901 | 0.797 | 0.884 | 0.864 |  |
|  | **2016 vs 2019** | | | | |
|  | 0.368 | 0.449 | 0.367 | 0.338 | 0.230 |

Supplementary Table 3.

Spearmann correlation coefficients for the RSA indices. The upper part presents coefficients for different variables in 2016. the middle part – for different variables in 2019. and the lower part – correlations of the same parameter measured at both visits. All correlations were statistically significant (p<0.05).
