# Supplementary material for: Respiratory Sinus Arrhythmia in Children—Predictable or Random?
Source: Front Cardiovasc Med. 2021 May 20;8:643846. doi: 10.3389/fcvm.2021.643846 (PMC8172810; doi:10.3389/fcvm.2021.643846)

A scatter chart showing ΔRR (pvRSA) at both visits.

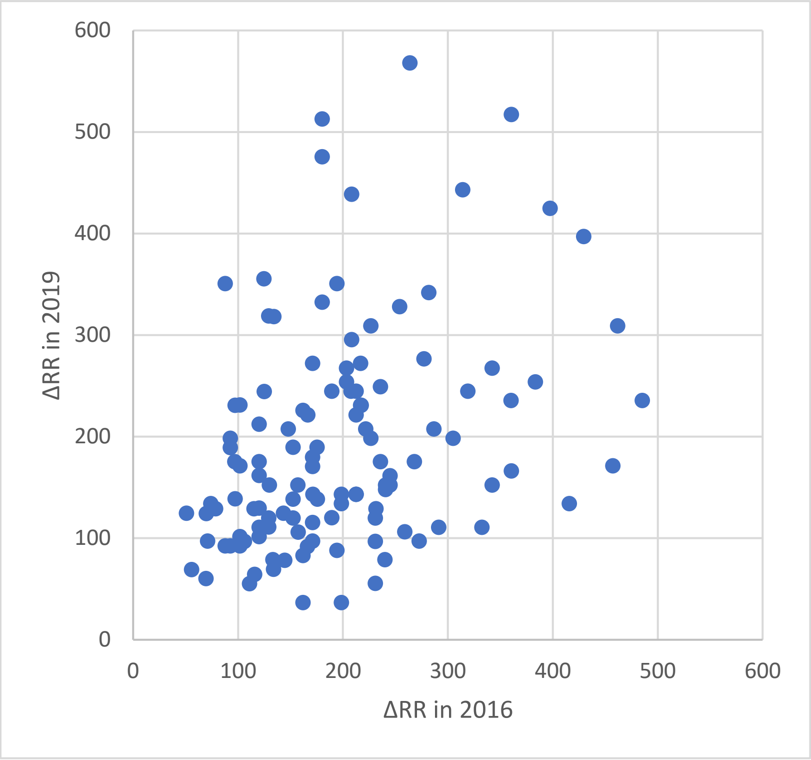

An example of supplementary figure to facilitate the comprehension of ring-graphs

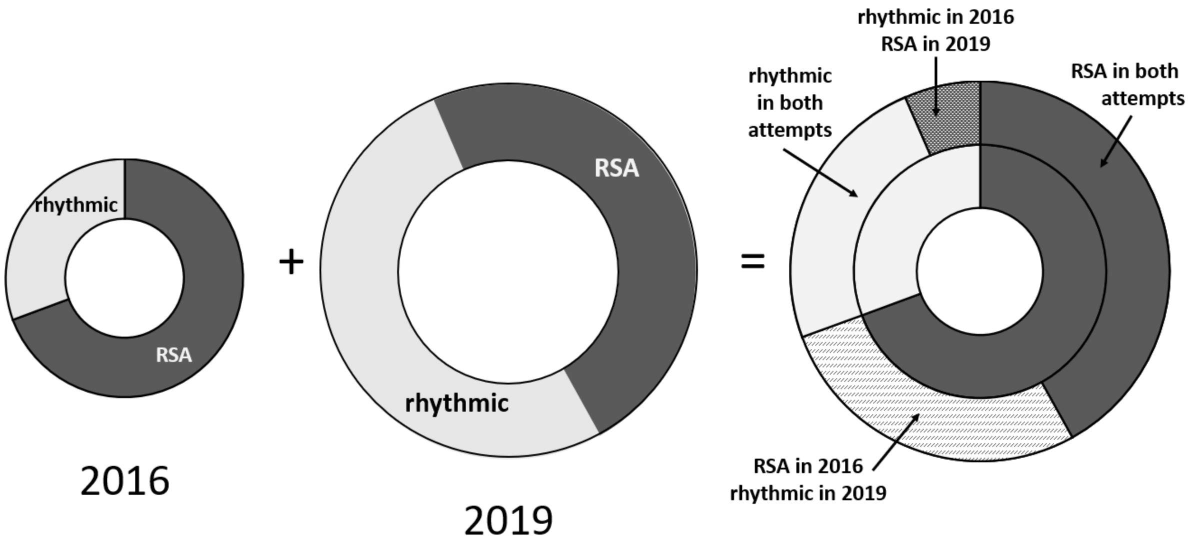

Supplement: Supplementary file 2 [file Data_Sheet_1.pdf]
